# Psychological risk profiles are associated with severity and motivational-volitional mechanisms of non-suicidal self-injury in adolescents and young adults

**DOI:** 10.3389/frcha.2026.1878269

**Published:** 2026-08-11

**Authors:** Elisa Schmied-Weggler, Zrinka Sosic-Vasic, Bernhard Connemann, Julia Kroener

**Affiliations:** 1 Department of Applied Psychiatry and Psychotherapy, Christophsbad Goeppingen, Goeppingen, Germany; 2 Department of Psychiatry and Psychotherapy III, University Clinic of Ulm, Ulm, Germany

**Keywords:** adolescence, cluster, emotion dysregulation, mental images, non-suicidal self-injury, NSSI, prospective intrusive mental imagery, self-efficacy

## Abstract

**Background:**

Non-suicidal self-injury (NSSI) is a prevalent behavior among adolescents and young adults. According to the Integrated Motivational–Volitional (IMV) model, pre-motivational vulnerabilities such as depression, anxiety, and low self-efficacy underlie self-harming behavior. It remains unclear how these factors combine into distinct clinical profiles and how such profiles differ regarding intrusive prospective mental imagery, emotion regulation, and NSSI frequency. Therefore, this study aimed to identify psychological subgroups based on distress and self-efficacy using a person-centered cluster approach, and to examine how these profiles, together with NSSI age of onset, relate to imagery intrusiveness, emotion regulation strategies, and NSSI frequency.

**Methods:**

Five hundred fifty-seven adolescents and young adults with a history of NSSI completed an online survey. A TwoStep cluster analysis based on depression (BDI-II), anxiety (STAI-T), and self-efficacy (WIRKALL_r) identified psychological profiles. General Linear Models (GLMs) with bootstrapping (1,000 resamples) examined the independent and interactive effects of cluster membership and NSSI age of onset (early: <12 vs. late: ≥12 years) on intrusive imagery (IFES-S), emotion regulation strategies (ERQ), and NSSI behavior (SITBI).

**Results:**

Two profiles emerged: a High-Risk cluster (high distress, low self-efficacy) and a Low-Risk cluster (low distress, high self-efficacy). The High-Risk cluster showed significantly higher NSSI frequency, greater intrusive imagery, increased suppression, and reduced cognitive reappraisal. Early NSSI age of onset was independently associated with higher lifelong NSSI frequency and greater imagery intrusiveness across both clusters. No significant cluster-by-onset interactions were found, supporting an additive risk model.

**Discussion:**

Findings support the pre-motivational phase of the IMV model and suggest that intrusive imagery and maladaptive emotion regulation align with the transition from distress to NSSI. The additive effects of psychological risk profile and early NSSI age of onset highlight the need for targeted interventions, including Imagery Rescripting and self-efficacy training.

## 1 Introduction

Non-suicidal self-injury (NSSI) is defined as the deliberate, direct destruction of one's own body tissue without suicidal intent and for reasons not socially sanctioned (1). NSSI is highly prevalent among adolescents and young adults (lifetime rates 17%–22%) (2–4), typically peaking in mid-adolescence (5). NSSI is frequently used as a maladaptive strategy to regulate intense negative emotional states (6) and is closely linked to psychological distress (7, 8). Early onset of NSSI (defined as ≤12 years) has been associated with more severe patterns of self-injurious behaviors, including higher frequency, greater method versatility, and injuries of greater medical severity (2). NSSI is also one of the strongest predictors of future suicidal behavior, even exceeding prior suicide attempts in its prognostic value (9), particularly when the onset occurs at a younger age (2). Despite growing clinical recognition, treatment options for NSSI remain limited in accessibility and effectiveness (10, 11).

Despite the heterogeneity of NSSI, integrative theoretical frameworks remain underutilized. The Integrated Motivational-Volitional (IMV) model (12) though originally developed for suicidal behavior, offers a robust framework for examining NSSI across pre-motivational, motivational, and volitional phases (12, 14). Its tripartite structure captures how affective dysregulation, the core function of NSSI, is generated by motivational factors and linked to volitional mechanisms. Within the pre-motivational phase, three factors are of particular relevance: self-efficacy, depression, and anxiety. Self-efficacy, defined as one's perceived capacity to regulate affect and manage stress (15), buffers this trajectory by modulating the impact of distress on the development of self-harming urges and by predicting NSSI onset (12, 13, 16). Anxiety and depression, conversely, represent significant affective diatheses that heighten an individual's negative reactivity to stress (12) and sensitize individuals to interpret stressors as defeat, thereby establishing the cognitive-affective context for subsequent NSSI urges and action. By focusing on these mechanisms, we can examine how individual “psychological risk profiles” are associated with different stages from distress to self-injury. In the present study, a “psychological risk profile” is operationally defined as the distinct cluster formed by the individual's distress level, self-efficacy, and NSSI age of onset.

**Figure 1:**
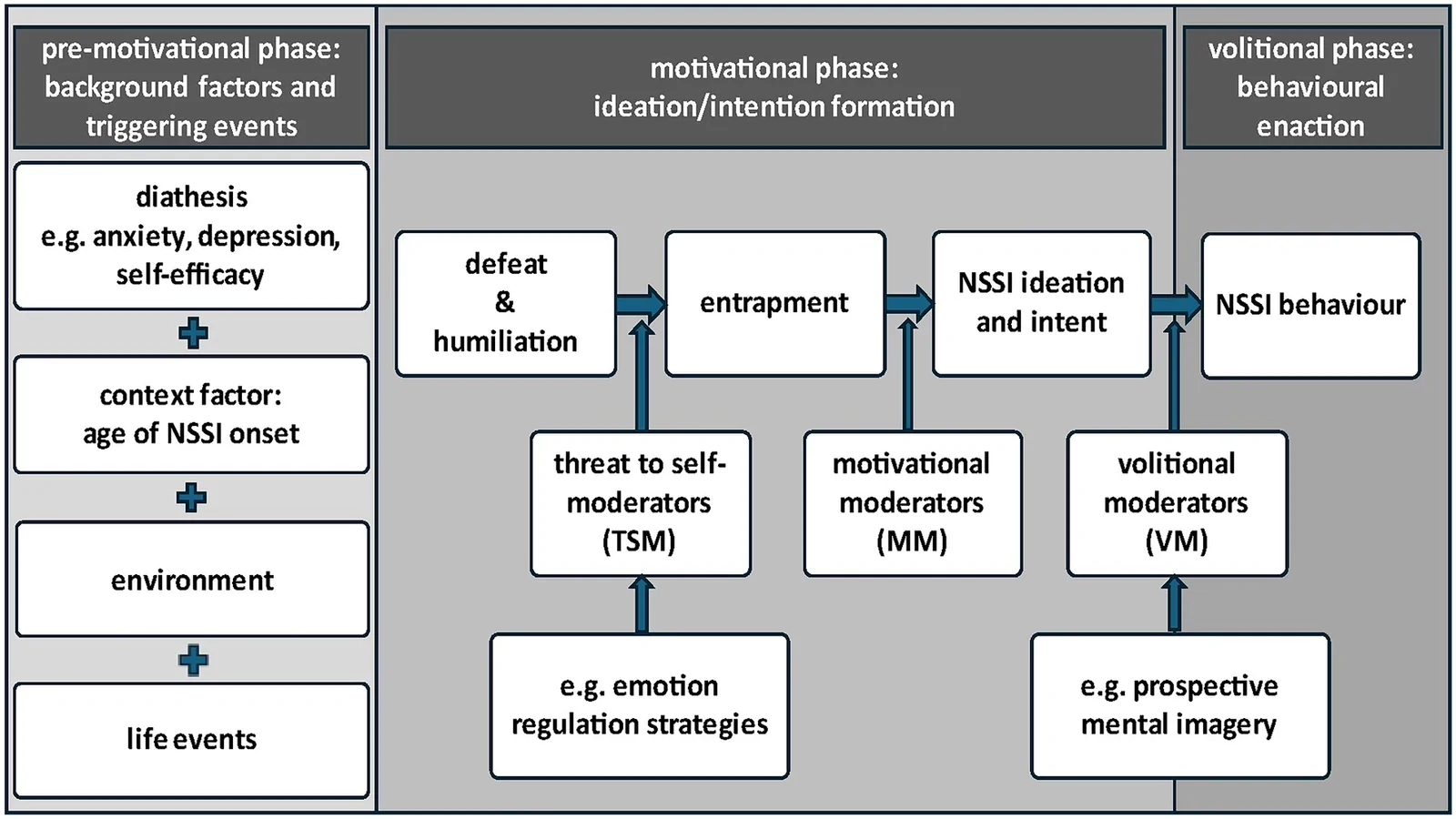
The integrated motivational–volitional (IMV) model of non-suicidal self-injury. Adapted from “The IMV model of suicidal behaviour” by Rory C. O'Connor and Olivia J. Kirtley, licensed under CC BY 4.0.

When individuals experience such defeat and the resulting intense negative affect (12, 13, 17), their capacity to cope with this internal distress is determined by their emotion regulation strategies. Adaptive strategies, such as reappraisal, act as “Threat-to-Self” moderators (12), allowing the individual to manage distress and mitigate the perceived inescapability of the situation. In contrast, maladaptive strategies, such as suppression, accelerate the transition from the painful experience of defeat to the inescapable sense of entrapment (18, 19) (see Figure 1).

Mental imagery has emerged as a relevant but under-examined cognitive-affective mechanism in the context of NSSI (20). Mental imagery, reported by up to 90% of individuals prior to NSSI (21, 22), reinforces the behavior through emotional alleviation or anticipated relief (23–25). Importantly, the model acknowledges the role of mental imagery in activating volitional processes as a volitional moderator. Empirical evidence supports the notion that imagery frequently precedes NSSI episodes and functions as a motivational amplifier (26). While the role of mental imagery has been explored in the context of suicidal ideation (27, 28), its contribution to NSSI, particularly among adolescents, remains insufficiently studied. Recent studies (26) found that over one-third of adolescents with NSSI report self-injury-related imagery. As individuals move from intention to action, such imagery functions as a key volitional factor in the transition to self-harming behavior.

While research on mental imagery and NSSI has increased during the past decade, integrative models capturing individual heterogeneity remain scarce. Traditional approaches often overlook how risk factors cluster within an individual to form a unique psychological profile. To address the psychological risk, person-centered methods like cluster analysis can identify clinically meaningful subgroups based on distinct psychological profiles (29–33). These different psychological risk profiles reduce the heterogeneity of self-harming adolescents and improve a differentiated understanding of the underlying mechanisms and their interaction at the individual level, which helps to prepare efficient treatment and prevention strategies.

The present study employs this approach in adolescents and young adults (aged 15–24) with recent NSSI (≥5 episodes/year) to identify profiles across the IMV stages. We hypothesize that a vulnerable pre-motivational profile (high anxiety, depression, low self-efficacy) will associate with maladaptive motivational mechanisms (dysfunctional emotion regulation) and heightened volitional factors (vivid NSSI-related imagery), ultimately correspond with a higher NSSI frequency.

## 2 Methods

### 2.1 Participants

A total of 557 adolescents and young adults participated in the online survey. Inclusion criteria required a minimum of five NSSI episodes within the past 12 months and the presence of intrusive prospective mental imagery related to self-harm. No exclusion criteria were applied.

Recruitment was conducted through multiple channels, including social media platforms (e.g., Instagram, Facebook), mailing lists, printed flyers, and the websites of the University Hospital of Ulm and the Christophsbad Clinic in Goeppingen, Germany. The study was advertised as an anonymous survey on self-harming behavior. Interested individuals accessed the questionnaire via a link provided through the SoSci Survey platform (34). At the end of the survey, participants had the option to submit contact information for potential participation in future studies. To enhance motivation, participants were informed that they would be entered into a raffle to win a €300 expense allowance. No additional compensation was offered.

### 2.2 Design and procedure

Participants accessed the study via a link, which directed them to a landing page outlining the study's aims, eligibility criteria, and conditions for participation. Those who did not meet the inclusion criteria were automatically exited from the survey and received a brief notice of ineligibility. Eligible participants proceeded to complete the questionnaire, which included a mix of open-ended items, single- and multiple-choice questions, and validated self-report measures using Likert scales (detailed in the Measures section). The average completion time was approximately 20 min. The survey was designed to be accessible on both desktop and mobile devices, allowing participants to complete it in a private setting of their choice. A progress bar displayed completion status throughout. Informed consent was obtained electronically prior to participation, and contact information was provided for questions or support. This study involved minor participants. Participation in the study was voluntary and fully anonymous, and therefore no written informed consent from parents or legal guardians was obtained. In accordance with German legal standards, the capacity of a minor to provide informed consent is assessed based on their mental and moral maturity. The Federal Court of Justice has ruled that minors aged 14 and above are generally presumed to have sufficient understanding to consent to medical or psychotherapeutic procedures (35). Furthermore, under §36 of the German Social Code Book I [SGB I], individuals aged 15 and above are considered legally competent in matters concerning social rights and benefits (35).

Based on this legal framework and in consultation with the institutional review board of the University of Ulm, Germany, we included participants aged 15–24 years (36). This study followed the Declaration of Helsinki. To safeguard participants, especially in light of sensitive topics such as suicidal ideation, we provided mental health support resources during and at the end of the survey. Participants were given emergency contact information as well as the contact details of the authors in case of distress.

### 2.3 Study flow

**Figure 2:**
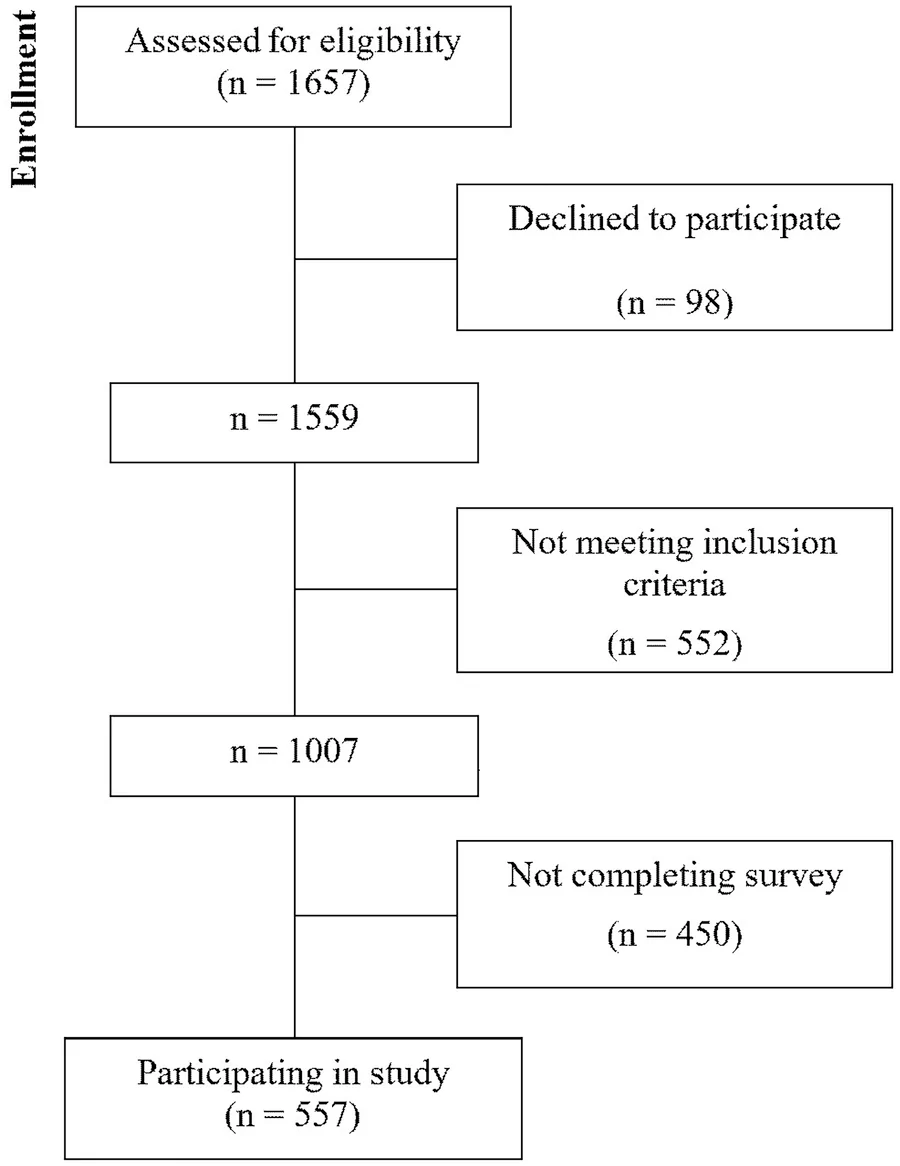
CONSORT flowchart – recruitment German online survey.

Participant's recruitment took place between August 2021 and May 2023. During this period, 1,657 individuals accessed the survey link and provided informed consent. Of these, 98 did not proceed beyond the consent page, 522 did not meet all inclusion criteria of the study and 450 did not complete the survey. The final sample consisted of 557 participants who met all eligibility requirements and completed the questionnaire. Please see Figure 2 for the CONSORT flowchart.

### 2.4 Measures

#### 2.4.1 Questionnaires

##### 2.4.1.1 NSSI

DSM-5 criteria (17, 37) was applied for delineating NSSI. The DSM-5 criteria require individuals to have experienced a minimum of five self-harming incidents within the previous year, with NSSI events occurring in the context of interpersonal difficulties or adverse emotional states, accompanied by recurrent thoughts of NSSI. This frequency threshold is proposed for NSSI Disorder in the DSM-5 (Section 3).

Furthermore, various items of the structured “Self-Injurious Thoughts and Behaviors Interview” (SITBI; (38)) were utilized to assess NSSI. The SITBI consists of 169 items, divided into five sections: Onset, Frequency, Methods, Functions, and Social Influences of NSSI. For the purpose of our study, we utilized the following items to assess NSSI: Item 117 (age onset about thoughts about NSSI), Item 144 (age onset of NSSI) Item 146 (frequency of NSSI lifetime), Item 147 (frequency of NSSI within a year), Items 152–156 (motives for NSSI), 164 (time between thoughts about NSSI and NSSI behavior).

##### 2.4.1.2 Emotion regulation

The Emotion Regulation Questionnaire [ERQ; (39, 40)] is a 10-item self-report questionnaire that measures two distinct strategies for managing emotions: cognitive reappraisal and expressive suppression. Items are rated on a 7-point Likert scale ranging from 1 (strongly disagree) to 7 (strongly agree). Within the current study, the ERQ demonstrated acceptable internal consistency (Cronbach's *α* = .73 for reappraisal, Cronbach's *α* = .65 for suppression).

##### 2.4.1.3 Emotional distress

The Beck Depression Inventory – Second Edition (BDI-II; (41)) is a 21-item self-report scale that assesses depressive symptoms over the course of the past two weeks, including the assessment day. The items are rated on a 4-point Likert scale from 0 (no symptoms) to 3 (severe symptoms), with total scores ranging from 0 to 63. The BDI-II demonstrated very good internal consistency in the present study (Cronbach's *α* = 0.88).

The State-Trait Anxiety Inventory – Trait Version (STAI-T; (42)) examines anxiety as a trait, specifically as a reasonably stable personality characteristic (trait anxiety), which denotes individual variations in the propensity to react to feared stimuli. The trait scale consists of 20 items rated on a 4-point Likert scale from 1 (almost never) to 4 (almost always). In the current study, the internal consistency was good (Cronbach's *α* = 0.86).

##### 2.4.1.4 Self-efficacy

General Self-efficacy was assessed via the Scale for General Self-efficacy Expectation (WIRKALL_r; (43)). The questionnaire includes 10 items evaluating participants’ beliefs in their ability to handle various challenging life situations, like “even when unexpected events occur, I believe that I will be able to handle them well” or “when I encounter a problem, I usually have several ideas about how to solve it”. Items are rated on a 4-point Likert scale from 1 (not at all) to 4 (very). In the current sample, the scale displayed good internal consistency (Cronbach's *α* = 0.81).

##### 2.4.1.5 Intrusive prospective mental imagery

The Impact of Future Events Scale – Short Version (IFES-S; (44, 45)) is a 24-item self-report scale, that assesses intrusive prospective imagery. To fit the scope of the current study, the instructions of the questionnaire was slightly modified: Participants were instructed to answer the questionnaire thinking about prospective intrusive images about self-harm and indicate how frequently each statement applied to them in the past seven days. Responses were given on a 5-point Likert scale ranging from 0 (not at all) to 4 (extremely). The IFES-S demonstrated very good internal consistency in the current sample (Cronbach's *α* = 89).

### 2.5 Statistical analysis

All statistical analyses were conducted via IBM SPSS Statistics (Version 28; (46)). Descriptive statistics, including absolute and relative frequencies, means, medians, and standard deviations, were calculated to characterize the sample, and the phenomenology of mental imagery.

To investigate patterns across psychopathology-relevant domains, we employed a TwoStep cluster analysis as the primary analytic approach using participants’ z-standardized scores for depression (BDI-II), anxiety (STAI-T), self-efficacy (WIRKALL_r). The optimal number of clusters was determined automatically based on the Bayesian Information Criterion (BIC).

For the purpose of solution robustness and validation, we conducted two additional sensitivity analyses. First, we performed a hierarchical agglomerative cluster analysis (Ward's method, squared Euclidean distance) using only the three continuous variables of the pre-motivational phase to guide and explore the number of clusters. Second, we performed a k-means analysis with the final predetermined cluster number to refine cluster membership. To validate the distinctness of the resulting clusters, Welch's ANOVAs were conducted for the clustering variables. This robust procedure was chosen to account for unequal group sizes and potential violations of the homogeneity of variance assumption.

Finally, the clusters were then used for multivariate General Linear Models (GLMs) to examine its effects on several outcome variables, including NSSI over the past year (SITBI), NSSI lifetime (SITBI), intrusive future-oriented imagery (IFES-S), as well as emotion regulation (ERQ).

To investigate the interplay between the identified clusters and the timing of NSSI age of onset, GLM were conducted using a 2 × 2 factorial design. The NSSI age of onset was transformed into a binary variable. The factors included cluster (cluster 1 vs. cluster 2) and NSSI age of onset (early onset: < 12 years vs. late onset: ≥ 12 years). This approach allowed for the simultaneous assessment of main effects for both factors as well as their potential interaction (cluster × NSSI age of onset) on clinical and psychological outcomes.

Preliminary assumption testing revealed significant violations of multivariate and univariate normality (as indicated by Q-Q plots and Kolmogorov–Smirnov tests) and a lack of homoscedasticity (Box's M test: *p* < .001; Levene's test: *p* < .05). Consequently, to ensure robust parameter estimates and valid statistical inference, a bootstrapping approach with 1,000 resamples was applied. Significance was determined based on 2-tailed *p*-values and 95% percentile bootstrap confidence intervals (CI). Effects were considered statistically significant if the 95% CI did not include zero. Partial eta-squared (*η*_p_^2^) was calculated as a measure of effect size.

## 3 Results

### 3.1 Sample characteristics

The majority of the 557 participants were female (430 females (77, 2%), 44 males (7, 9%), and 83 individuals with varied gender identities (14, 9%)). The age ranged from 15 to 24 years (mean = 19.7, SD = 2.37). In terms of current educational attainment, 1.8% reported not pursuing any degree (*n* = 10), 3.8% were pursuing a lower secondary school degree (in German: Hauptschulabschluss, equivalent to Grade 9 certificate; *n* = 21), 18.9% were working towards an intermediate secondary school certificate (in German: Realschulabschluss; *n* = 105), 51.5% were preparing for their high school diploma (in German: Abitur/Fachhochschulreife; *n* = 287), and 24.1% were enrolled in university, working towards a bachelor's or master's degree (*n* = 134). With respect to the occupational status, 29.8% were still in school (*n* = 166), 13.6% were in vocational training (*n* = 76), 25.5% were university students (*n* = 142), and 8.1% were currently employed (*n* = 45). Additionally, 5.6% were unemployed (*n* = 31), 16.9% were on medical leave (*n* = 94), and 0.5% were early retirees (*n* = 3).

Concerning therapeutic experience, 4.3% were on a waiting list to receive outpatient treatment (*n* = 24), 24.1% had previously received therapeutic treatment (more than three months ago; *n* = 134), and 58.2% were in treatment at the time of the study (*n* = 324), whereas 13.5% have never been in therapy (*n* = 75). Moreover, 62% (*n* = 344) of participants reported having attended at least one inpatient treatment, while 17.8% (*n* = 213) had never done so. Finally, 82.3% (*n* = 478) reported having received outpatient treatment, whereas 17.8% (*n* = 99) had not received such treatment. When asked whether they had ever sought help for NSSI, 20.1% said no (*n* = 112). Among those who had sought help, 5.7% used online resources (*n* = 32), 4.3% contacted a counselling center (*n* = 24), 3.9% consulted a general practitioner (*n* = 22), 52.4% received support from a psychotherapist (*n* = 292), and 13.5% from a psychiatrist (*n* = 75).

Most participants (80%) had already sought help concerning NSSI in the past. Those participants who had not sought help for NSSI (*n* = 112) cited the following reasons: 21.4% stated that they could manage on their own (*n* = 24), 13.4% reported feelings of shame (*n* = 15), 8% did not believe help would be effective (*n* = 9), 22.3% feared that their parents might find out (*n* = 25), 7.1% reported a lack of time (*n* = 8), and 27.7% gave other reasons (*n* = 31). Other reasons included the barrier to communicate with others out of fear or insecurity whom to tell, as well as not doing bad enough, see Table 1. Finally, 51.3% of the sample reported current use of psychotropic medication (*n* = 286), whereas 48.7% (*n* = 271) did not. For the distribution of diagnoses see Table 2.

**Table 1:** Self-proclaimed reasons for no kind of assistance due to NSSI (each participant could proclaim more than one reason).

Reasons	No
Afraid to tell it to somebody (e.g., parents/not to be taken seriously)	8
I am not doing bad enough/ others are doing worse	7
Too difficult to find a therapist	3
No trust in therapists/doctors	4
I do not deserve it	2
I do not want to stop/it helps me/I do not see it as a problem	5
Other things are more important	3
Anxiety disorder	1
Afraid of having this in my medical record	1

**Table 2:** Self-reported diagnoses by participants.

Diagnoses	%
Depression/Dysthymia	26
Anxiety disorders (Agoraphobia, panic disorder, general anxiety, social phobia, specific phobia, separation anxiety, mixed anxiety and depressive disorder)	15.2
Personality disorder (Persistent personality change after extreme stress, borderline, insecure, combined, anankastic)	18.6
(Complex) PTSD	11.3
Eating disorder (Anorexia nervosa, bulimia nervosa, eating disorder)	8.8
AD(H)D	3.5
Others	7.2
Without diagnoses	9.5

Note: AD(H)D, attention deficit (hyperactivity) disorder; PTSD, posttraumatic stress disorder.

### 3.2 Occurrence and characteristics of NSSI (-images)

Mental imagery related to NSSI rarely pertained exclusively to either past (*n* = 63) or future events (*n* = 79). The vast majority of participants (*n* = 415) reported experiencing hybrid imagery, comprising of both, autobiographical memories and anticipated future scenarios. On average, participants reported the onset of NSSI-related thoughts at age 11.9 years (SD = 3.0), with the first engagement in NSSI occurring at approximately 12.5 years (SD = 3.1).

When asked about their primary motivations for engaging in NSSI, 65.2% (*n* = 363) indicated emotion regulation, specifically, the alleviation of negative affect, as the most prominent reason. Furthermore, 50.4% (*n* = 281) endorsed engaging in NSSI to counter emotional numbness (“to feel something”), and 49.4% (*n* = 275) cited self-punishment as a central motivation. Yet, only 8.8% (*n* = 49) agreed strongly with the motive of using NSSI as a form of communication or to draw attention.

The average latency between the onset of NSSI-related thoughts and NSSI conduct was approximately five minutes (*M* = 5.34; *SD* = 2.0). Finally, when asked to estimate the probability of engaging in NSSI in the future, participants reported an average likelihood of 80.01% (*SD* = 26.6).

### 3.3 Cluster analysis

The K-means algorithm divided the sample into two distinct groups. The subsequent robust ANOVAs revealed highly significant differences between the clusters across all dimensions (*p* < .001). Cluster 1 (High Distress): Characterized by significantly elevated scores in anxiety (*F*_Welch_ (1, 319.13) = 524.02, *p* < .001, *η*^2^=.53) and depression *F*_Welch_ (1, 352.63) = 714.84, *p* < .001, *η*_2_=.59). Cluster 2 (Resilient/Low Distress): Displayed significantly higher levels of self-efficacy (*F*_Welch_ (1, 402.64) = 174.26, *p* < .001, *η*^2^=.25) compared to Cluster 1. These results underscore a clear dichotomy: Cluster 1 represents a high-distress profile (elevated anxiety and depression, lower self-efficacy), whereas Cluster 2 represents a resilient profile with significantly lower symptom levels and higher perceived self-efficacy. The substantial effect sizes, particularly for the distress variables, underscore the high discriminative power of the cluster solution, explaining over 50% of the variance in symptomatic distress through cluster membership.

### 3.4 General linear model analysis

The GLM revealed significant differences between Cluster 1 (*n* = 351) and Cluster 2 (*n* = 206) across all investigated variables.

**Figure 3:**
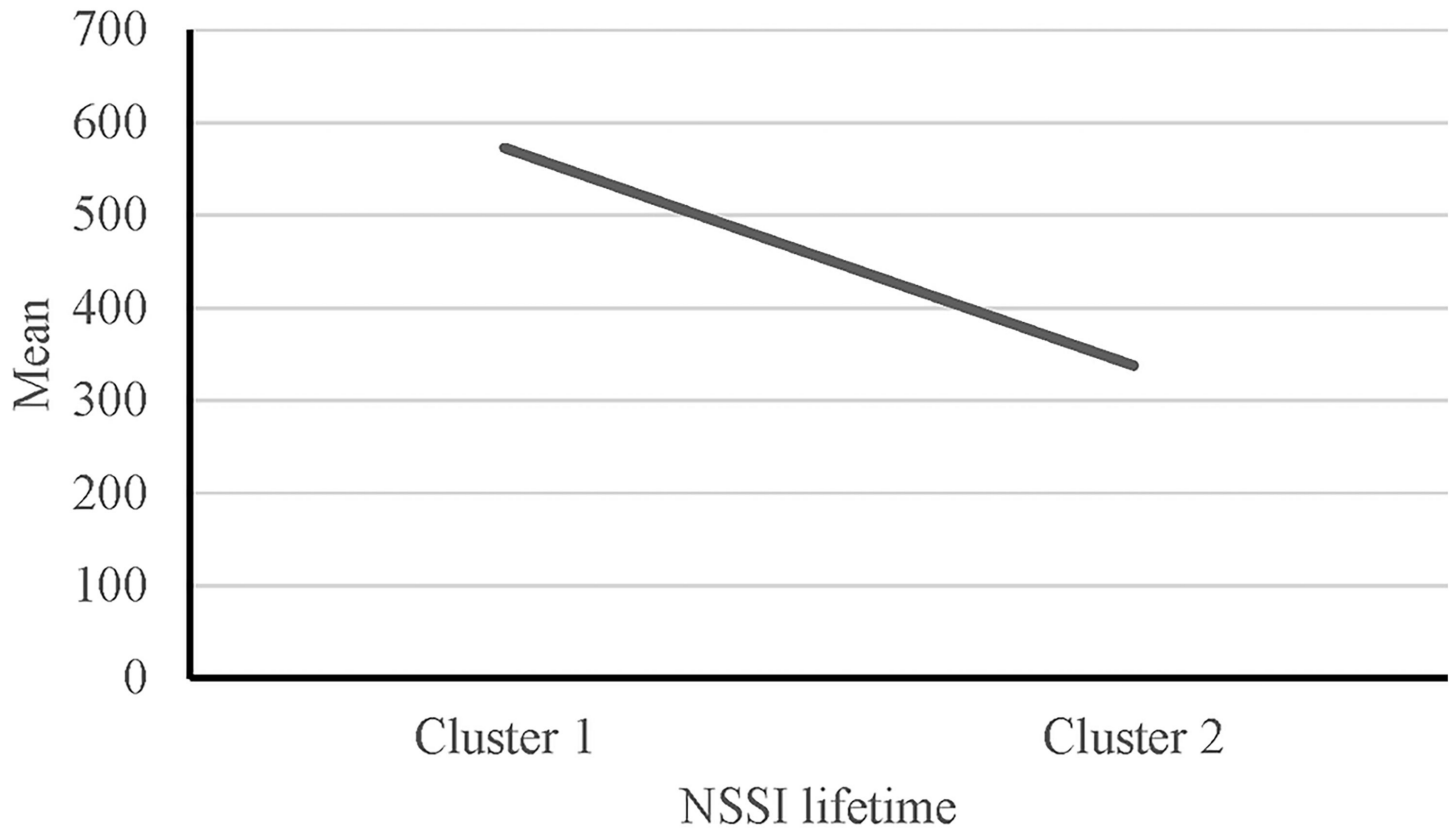
Comparison between clusters and NSSI outcome variables (NSSI lifetime). Note: NSSI = Non-suicidal self-injury, NSSI lifelong = NSSI frequency in the whole life span.

**Figure 4:**
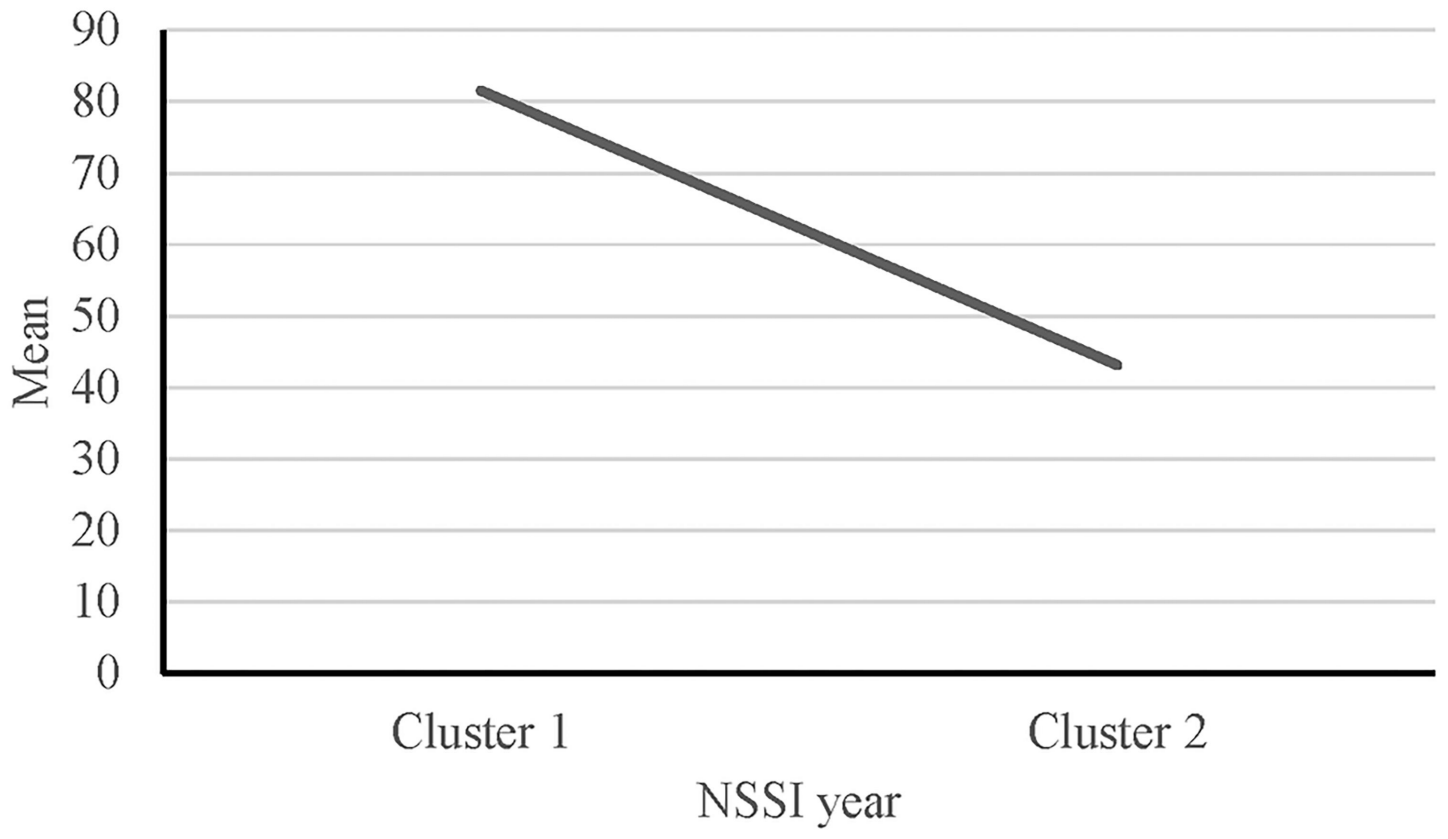
Comparison between clusters and NSSI outcome variables (NSSI year). Note: NSSI = Non-suicidal self-injury, NSSI year = NSSI frequency in the last 12months.

Regarding clinical symptoms, Cluster 1 exhibited significantly higher levels of lifetime NSSI (*F*(1, 555) = 9.53, *p* = .002, *η_p_*^2^=.017; Bootstrap *B* = 235.11, 95% CI [79.44, 373.86]) and a higher frequency of NSSI within the last year (*F*(1, 555) = 31.03, *p* < .001, *η_p_*^2^=.053; Bootstrap *B* = 38.41, 95% CI [25.17, 50.69]) compared to Cluster 2 (see Figures 3, 4).

**Figure 5:**
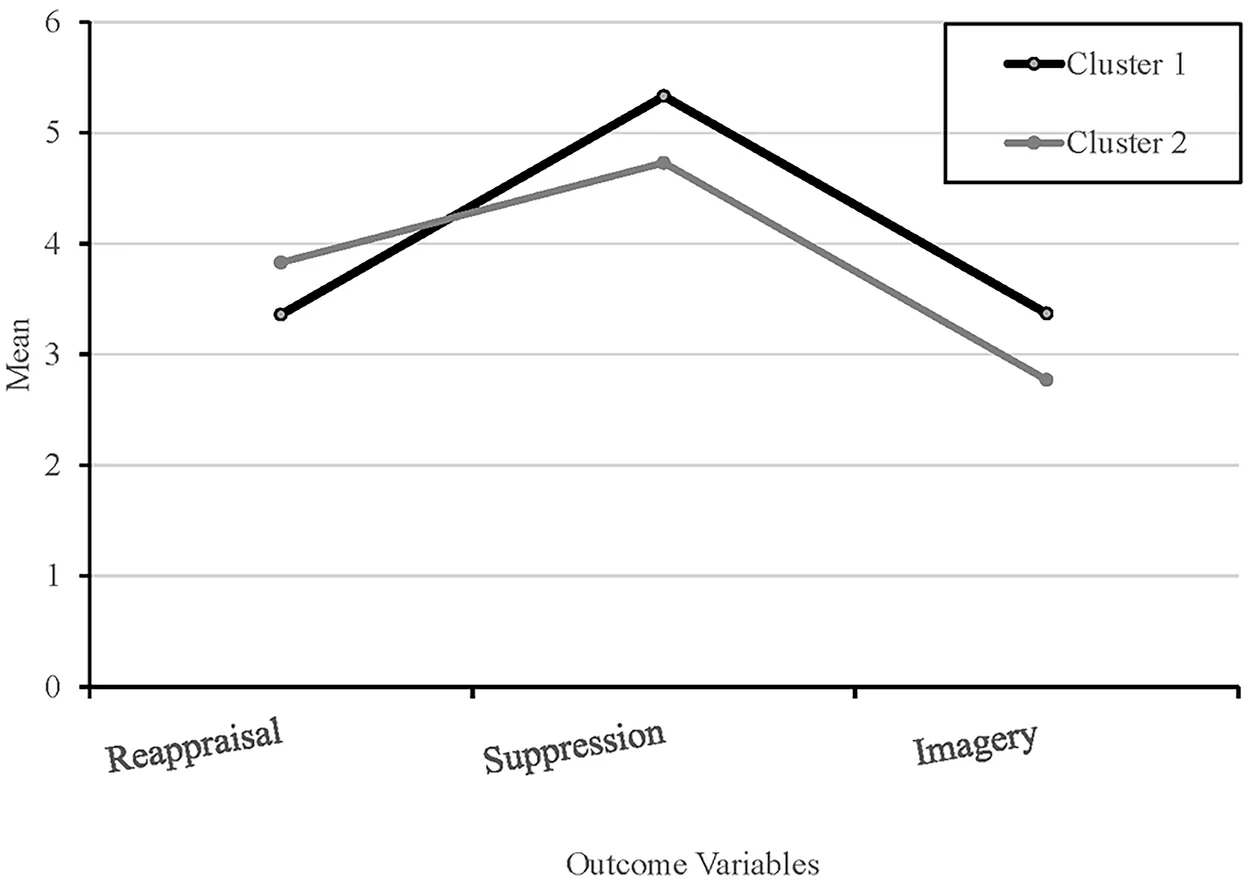
Comparison between clusters and outcome variables. Note: Imagery, prospective intrusive mental imagery.

In terms of emotion regulation, Cluster 1 showed significantly lower scores in reappraisal emotion regulation strategies (*F*(1, 555) = 20.56, *p* < .001, *η*_p_^2^=.036; Bootstrap *B* = −0.47, 95% CI [−0.68, −0.27]) but higher scores in suppression emotion regulation strategies (*F*(1, 555) = 30.02, *p* < .001, *η*_p_^2^=.051; Bootstrap *B* = 0.60, 95% CI [0.38, 0.83]). Furthermore, Cluster 1 was characterized by significantly higher scores on intrusiveness of prospective mental imagery (*F*(1, 555) = 126.76, *p* < .001, *η*_p_^2^=.186; Bootstrap *B* = 0.61, 95% CI [0.49, 0.72]). All findings remained robust under the bootstrapping procedure, confirming the distinct psychological profiles of the two identified clusters (see Figure 5).

### 3.5 Explorative cluster analysis, including NSSI age of onset

**Figure 6:**
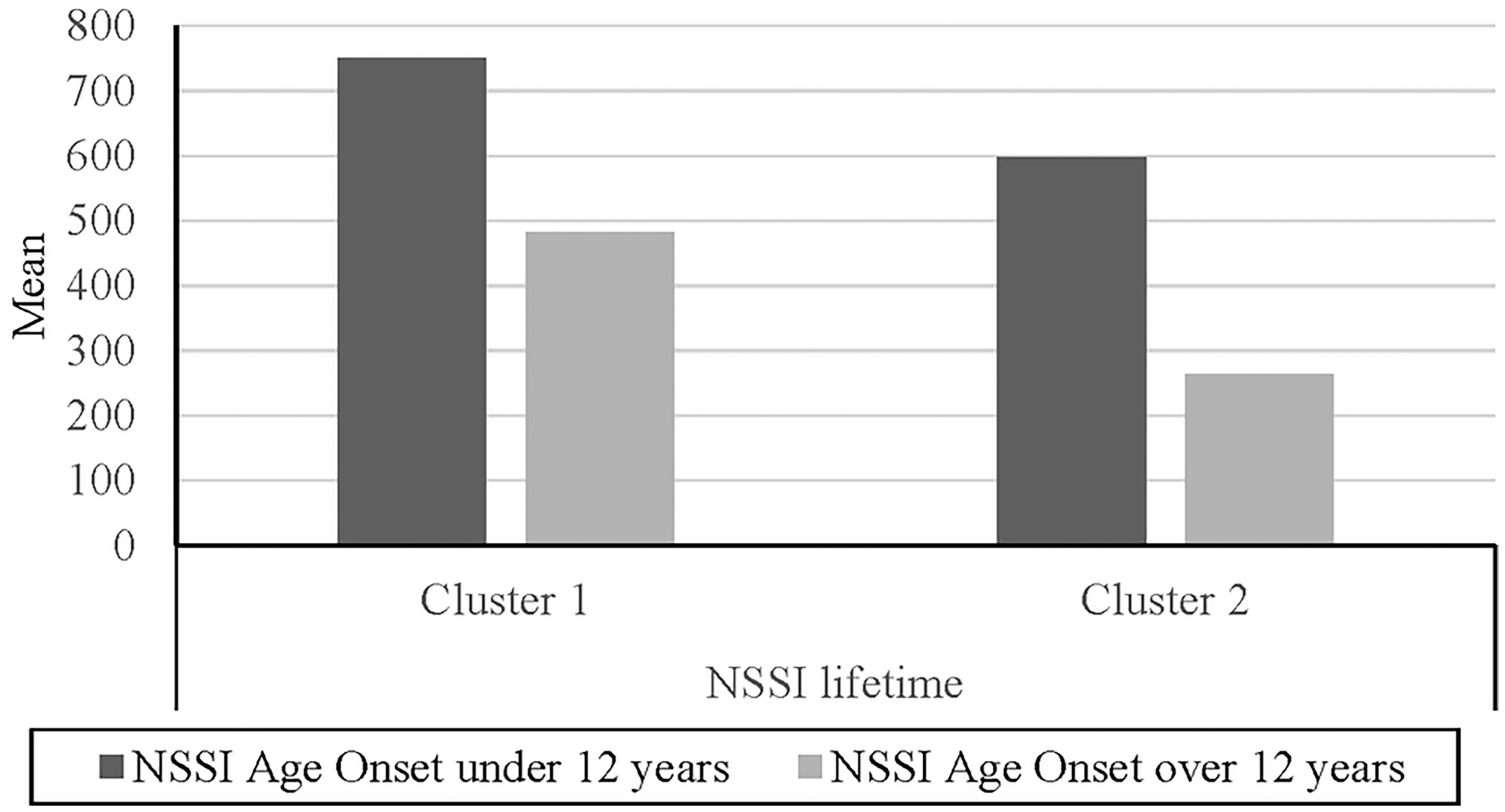
Comparison between clusters, NSSI age of onset and outcome variables (NSSI lifetime). Note: NSSI = Non-suicidal self-injury, NSSI lifelong = NSSI frequency in the whole life span.

**Figure 7:**
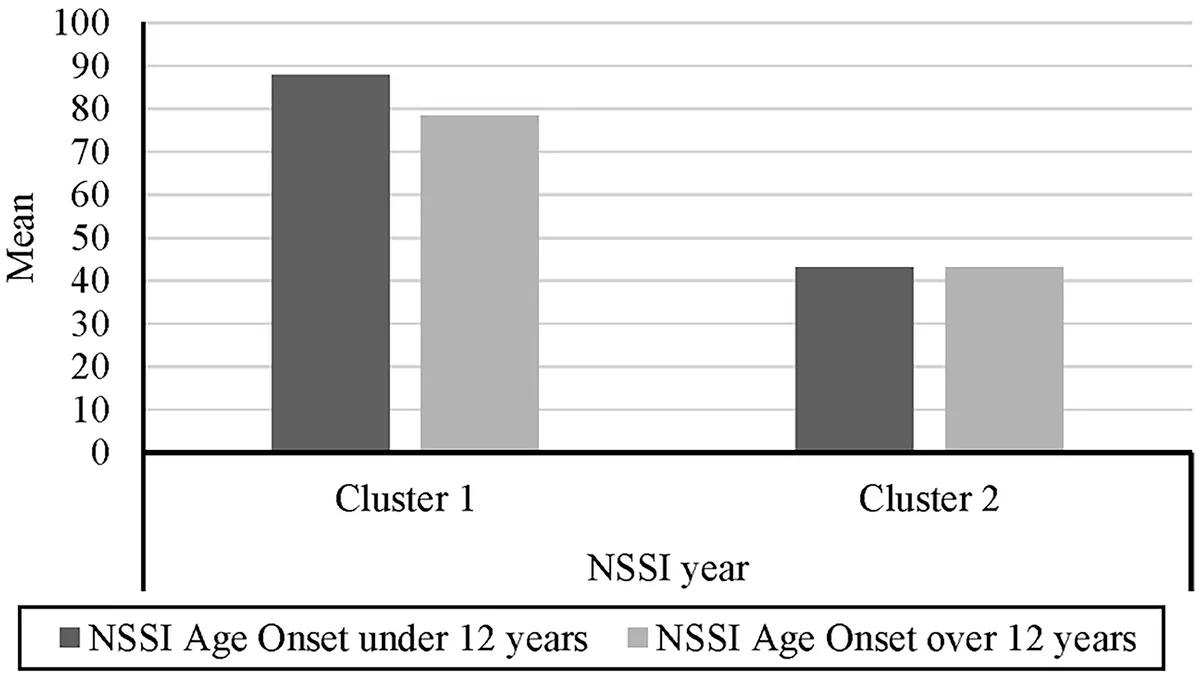
Comparison between clusters, NSSI age of onset and outcome variables (NSSI year). Note: NSSI = Non-suicidal self-injury, NSSI year = NSSI frequency in the last 12 months.

**Figure 8:**
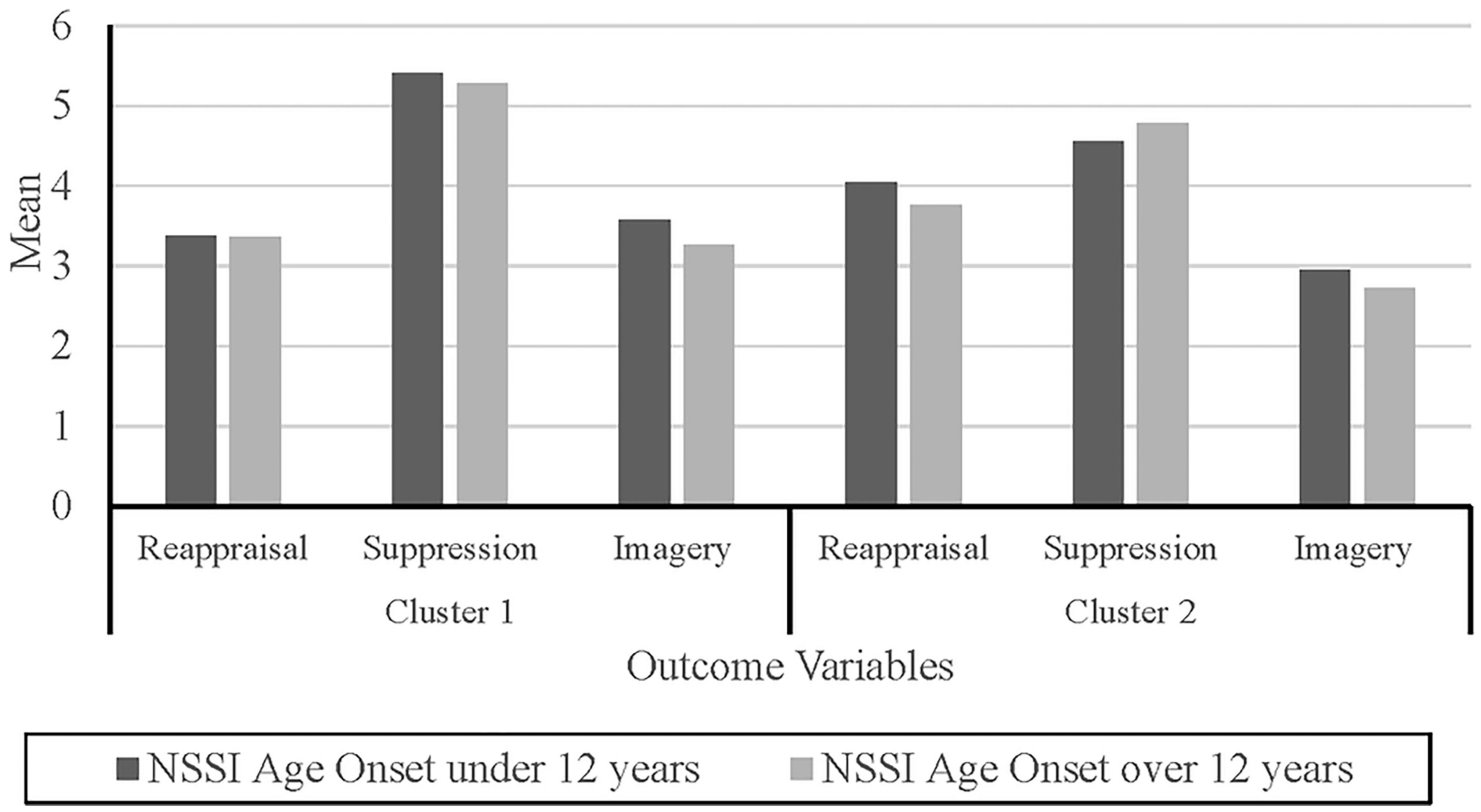
Comparison between clusters, NSSI age of onset and outcome variables. Imagery, prospective intrusive mental imagery.

The 2 × 2 GLM revealed significant main effects for both Cluster and NSSI age of onset. For lifetime NSSI, we found a significant main effect of cluster (*F*(1, 553) = 4.59, *p* = .033, *η*_p_^2^ = .008) and a significant main effect of NSSI age of onset (*F*(1, 553) = 12.19, *p* < .001, *η*_p_^2^=.022) (see Figures 6, 7). Importantly, the interaction between cluster and NSSI age of onset was not significant (*p* = .702), suggesting that early NSSI age of onset acts as an additional, independent risk factor. For intrusiveness of prospective intrusive mental imagery, the strongest effect was observed for cluster (*F*(1, 553) = 95.43, *p* < .001, *η*_p_^2^=.147), while NSSI age of onset also contributed significantly (*F*(1, 553) = 20.16, *p* < .001, *η*_p_^2^=.035). Again, no interaction was found (*p* = .524). For reappraisal emotion regulation strategies, cluster 1 showed significantly lower scores than cluster 2 (main effect cluster: *F*(1, 555) = 21.18, *p* < .001, *η*_p_^2^=.037) (see Figure 8). No significant effect of NSSI age of onset, *F*(1, 553) = 1.54, *p* = .215, or interaction, *F*(1, 553) = 1.37, *p* = .242, was found. For suppression emotion regulation strategies, both a significant main effect of cluster, *F*(1, 553) = 29.11, *p* < .001, *η*_p_^2^=.050, and a non-significant effect for NSSI age of onset, *F*(1, 553) = .10, *p* = .753, were tested, showing cluster 1 had higher levels. The interaction was not significant, *p* = .177. The strongest effects were observed for intrusiveness of prospective intrusive mental imagery. There was a large significant main effect of cluster, *F*(1, 553) = 95.43, *p* < .001, *η*_p_^2^=.147, and a significant main effect of NSSI age of onset, *F*(1, 553) = 20.16, *p* < .001, *η*_p_^2^=.035. Cluster 1 and individuals with early onset both exhibited significantly higher intrusiveness. The interaction effect remained non-significant (*F*(1, 553) = .41, *p* = .524).

## 4 Discussion

The present study reinforces the clinical utility of a person-centered approach in identifying meaningful subgroups of individuals engaging in NSSI. Although cluster analyses have been conducted before, the incorporated variables of self-efficacy and prospective intrusive mental imagery were previously underexplored in clustering studies. By integrating psychological distress, self-efficacy, and intrusive mental imagery, we identified two distinct clusters that reflect different vulnerability profiles within the pre-motivational phase of the IMV model. The identification of two distinct psychological clusters supports the framework of the IMV model, specifically its pre-motivational phase. In this stage, background factors and individual vulnerabilities, including depression, anxiety, and self-efficacy, form the psychological landscape that is associated with an individual's susceptibility to self-harming ideation. Cluster 1 presented a high-risk profile characterized by higher distress and lower self-efficacy. This constellation was associated with a more severe clinical trajectory, including higher lifelong NSSI frequency, more intense mental imagery, and the frequent use of maladaptive emotion regulation strategies such as expressive suppression (47).

These findings build on prior work linking anxious and depressive symptomatology to NSSI (29, 31, 48). Beyond mere symptom severity, the heightened psychological burden in Cluster 1 can be interpreted through the lens of Beck's cognitive triad (41), where a pervasive negative view of the self, the world, and the future creates a stable cognitive diathesis for self-harm. In the context of the IMV model, this triad may exacerbate the transition into the motivational phase by intensifying the experience of defeat. For adolescents in particular, this cognitive vulnerability meets a developmental period marked by identity formation, heightened academic pressure, and emotional instability (49). Under these conditions, the negative cognitive bias likely manifests as acute anxiety regarding social evaluation and a fear of interpersonal rejection. This fear of not meeting perceived external expectations or being socially excluded serves as a potent emotional stressor (50). If an individual concludes that their distress is both unbearable and unchangeable, a hallmark of the negative future expectations within the triad, the resulting sense of entrapment becomes a primary correlate of NSSI. In this state, self-injury is no longer just a behavior, but a regulatory attempt to escape an internal environment perceived as hopeless and confining. Specifically, higher sensitivity to social rejection is associated with more frequent negative imagery, often involving social situations, and a significant lack of control over these mental intrusions (51, 52). Thus, mental imagery does not merely reflect distress but acts as a cognitive-emotional amplifier which bridges the motivational state of entrapment to the volitional act of self-harm.

In our sample, self-efficacy emerged as a critical factor, consistent with prior findings that highlight its protective role (13, 53) within the pre-motivational background of the individual. Interestingly, our findings regarding the persistence of these profiles align with recent evidence (54), who documented a discrepancy between significant clinical improvement (NSSI reduction) and stable levels of general self-efficacy. This suggests that while behavioral change can be achieved, the global belief in one's coping capabilities, a core pre-motivational factor in the IMV model, is more resistant to change. This lag may be explained by the tendency of individuals to initially attribute behavioral success to external factors, such as therapeutic interventions or digital tools, rather than their own internal competence (55). For long-term recovery, it is therefore essential that interventions not only focus on behavioral cessation but specifically target the internal consolidation of self-efficacy. Most participants reported experiencing vivid and emotionally charged mental images related to both past and anticipated future self-harm events (56). These intrusions typically occurred shortly before NSSI behavior, with an average latency of five minutes. Most participants reported experiencing vivid, emotionally charged mental images of future self-harm, which may act as a cognitive-emotional amplifier within the IMV model (57). Such imagery may serve multiple purposes, including the alleviation of emotional distress, self-punishment, or the anticipation of relief, thereby reinforcing the behavior (23–25). By increasing motivational distress and accelerating the transition to action, these images represent a significant risk factor that was markedly more intense in the high-distress Cluster 1.

Additional calculations with the variable age of NSSI age of onset were conducted, due to the fact that early onset is widely recognized as a predictor of chronicity and poorer prognosis (2). We found that when age of onset was included directly in the TwoStep clustering, it acted as the dominant factor in partitioning the sample, essentially masking the independent contribution of the psychological symptom profiles. The statistical dominance of the age of onset in our clustering analysis underscores its role as a fundamental developmental marker. As other research (2) suggests, individuals with an early onset (≤12 years) represent a distinct risk group, reporting significantly higher lifetime frequencies even when the duration of the behavior is controlled for. This suggests that early-onset NSSI is not merely a matter of having “more time” to engage in the behavior, but rather indicates a process of habituation (58). Over time, individuals may habituate to the fear and pain associated with self-injury, requiring increased frequency or greater severity to achieve the same physiological or psychological regulatory effects. In the context of the IMV model, this habituation critically influences the volitional phase. Prolonged engagement in NSSI, may lead to the behavior becoming a “coping mechanism” (2, 58). While later-onset NSSI often follows an “experimental” pattern that is easier to consolidate, early onset leads to a reliance on self-injury that is harder to disrupt. Furthermore, this could intensify the intrusiveness of mental imagery. Through years of repetition, the mental representations of NSSI may become more deeply encoded and more easily triggered by distress. Crucially, our findings of a non-significant interaction between cluster and NSSI age of onset suggest an additive risk model. This implies that early onset does not change the fundamental nature of the psychological risk profiles but independently shifts the clinical trajectory toward higher chronicity and more intense imagery.

Notably, a substantial portion of the sample reported receiving simultaneous psychological treatment (58.2%) and utilizing psychotropic medication (51.3%). Despite this high rate of professional support, overall distress levels, intrusive imagery, and NSSI frequencies remained alarmingly high, particularly within the High-Risk cluster. This persistent symptom severity despite ongoing treatment highlights that standard care may not be sufficiently effective for all affected individuals. It strongly underscores our conclusion that clinical care must move beyond generalized approaches. Identifying distinct psychological risk profiles is a crucial step toward developing targeted, subgroup-specific interventions that address the unique mechanisms maintaining NSSI in these highly burdened patients.

Methodologically, while our approach combined multiple clustering algorithms (TwoStep, Ward's, k-means) to ensure high robustness and account for our specific sample, it relies on distance-based measures. This data-driven, bottom-up approach was explicitly chosen to maximize within-group homogeneity without imposing strict distributional assumptions (59). Although our sample size would generally be sufficient to yield stable estimates in probabilistic, model-based approaches like Latent Profile Analysis (LPA) (60, 61), this method was not applied in the current study. This is based on a recent systematic review on NSSI subgroups, demonstrating that different data-driven analytical approaches, including cluster analysis and LPA, generally derive similar subgroup typologies in terms of number and differentiating characteristics (62). Furthermore, obtaining the same results with LPA compared to the well-established conceptual and statistical overlap between depression (BDI-II) and anxiety (STAI-T) (63), would require explicit modeling residual covariances between indicators to avoid violating the local independence assumption. Without this additional modeling step, the risk of extracting artificial or spurious classes would increase. Therefore, our multi-step clustering approach provides stable and reliable group assignments for the given dataset. However, future studies should employ LPA to evaluate whether such model-based estimations yield similar latent profiles. Replicating our findings with mixture modeling would further validate the construct validity of the subgroups identified here.

To further explore whether these psychological profiles function independently of developmental timing, we employed a 2 × 2 factorial GLM. Consequently, we employed a 2 × 2 factorial GLM to disentangle these effects. The lack of a significant interaction effect suggests an additive risk model: early onset does not change the nature of the clusters but rather shifts the clinical profile toward a more severe trajectory. For clinicians, this highlights that adolescents and young adults with an early NSSI onset require intensified screening, particularly given the established links between early-onset NSSI and later suicidality (2, 9), even if their general psychopathological profile appears less severe at first glance. The findings underscore the clinical importance of addressing both current psychological distress and developmental history. If intrusive, distressing images can be identified early, they may serve as critical intervention points to prevent escalation. Imagery-based techniques, such as Imagery Rescripting (IR), have demonstrated preliminary efficacy in reducing NSSI and enhancing self-efficacy (64–67). Addressing deficits in emotion regulation, such as increasing the use of reappraisal over suppression, may further enhance treatment outcomes for those in the high-risk group.

## 5 Limitations

Several limitations should be considered. First, recruitment via social media and clinic websites may limit the generalizability of the findings to the broader NSSI population. The sample was predominantly female, which, while consistent with epidemiological trends, limits insights into male and non-binary individuals (68, 69). Furthermore, the study relied on self-reported diagnoses rather than structured clinical interviews, which may lack clinical confirmation. In addition, our inclusion criteria required a minimum of five NSSI episodes in the past year, aligning with the frequency threshold proposed for NSSI Disorder in the DSM-5 (Section 3). While this ensures a sample with clinically significant behavior, it excludes individuals with lower-frequency self-harm. Research suggests that these individuals may represent a distinct subgroup with different psychological drivers (30, 32) and by focusing on higher-frequency behavior, our findings may primarily apply to those meeting the full diagnostic threshold rather than the broader spectrum of self-injurious behavior.

Furthermore, while we recorded current therapeutic and pharmacological status, we did not assess the specific modality, adherence, or duration of these interventions. Future longitudinal studies should systematically control for these treatment variables to better understand their influence on the observed psychological profiles. Participation was restricted to individuals aged 15 and above, yet many participants reported NSSI onset around age 12. Future studies should investigate younger adolescents and track the development of NSSI and related imagery over time.

## 6 Conclusions

This study is among the first to cluster variables such as self-efficacy, additionally NSSI age of onset was conducted. The resulting subgroups differed in NSSI behavior, emotion regulation strategies, and intrusive prospective mental imagery in adolescents with NSSI. These findings underscore the heterogeneity of NSSI and the value of person-centered methods in identifying clinically relevant subtypes.

Aligned with the IMV model (12), our results highlight the importance of emotional distress and self-efficacy as vulnerability factors. Vivid, emotionally charged imagery may act as a catalyst for self-harm, particularly in those with limited coping resources. Interventions that target these processes, including imagery-based therapies and strategies to enhance self-efficacy, may offer promising avenues for prevention and treatment.

Future research should further explore the role of NSSI age of onset as a covariate in the model. Furthermore, the role of intrusive prospective mental imagery within the IMV framework should be focused to evaluate the efficacy of brief, targeted interventions, such as IR, that disrupt the progression from ideation to self-harming behavior in adolescents at risk. Future research should specifically investigate whether individuals in the High-Risk cluster (characterized by high distress and low self-efficacy) benefit disproportionately from such imagery-based techniques compared to more resilient profiles. Identifying which subgroup shows the highest responsiveness to these interventions would allow for a more personalized and resource-efficient approach in clinical settings.

## Data Availability

The raw data supporting the conclusions of this article will be made available by the authors, without undue reservation.
